# Antioxidant Enzyme Responses Induced by Whiteflies in Tobacco Plants in Defense against Aphids: Catalase May Play a Dominant Role

**DOI:** 10.1371/journal.pone.0165454

**Published:** 2016-10-27

**Authors:** Haipeng Zhao, Xia Sun, Ming Xue, Xiao Zhang, Qingliang Li

**Affiliations:** 1 College of Plant Protection, Shandong Agricultural University, Tai’an, Shandong, China; 2 Shanxi Academy of Agricultural Sciences, Jinzhong, Shanxi, China; Gyeongnam National University of Science and Technology, REPUBLIC OF KOREA

## Abstract

**Background:**

*Bemisia tabaci* MEAM1 (Middle East-Asia Minor 1) feeding alters antioxidative enzyme activity in some plant species. Infestation of *B*. *tabaci* nymphs decreases *Myzus persicae* performance on systemic, but not local leaves of tobacco plants. However, it is unclear if *B*. *tabaci* nymphs induced antioxidant activities contributing to the aphid resistance.

**Methodology/Principal Findings:**

We investigated the relationship between antioxidants induced by nymphs of *B*. *tabaci* feeding on tobacco and aphid resistance. The activities of catalase (CAT), peroxidase (POD), superoxide dismutase (SOD) and the concentration of hydrogen peroxide (H_2_O_2_) were assayed in tobacco leaves at different feeding times following infestation of *B*. *tabaci* nymphs. The infestation altered the activities of CAT and POD, but had no significant effect on SOD activity. The highest CAT activity was observed at 15 d after infestation. This was 98.2% greater than control systemic leaves, but 32.6% lower than the control in local leaves. Higher POD activity was recorded in local vs. systemic leaves after 15 d of infestation. POD activity was 71.0% and 112.9% higher in local and systemic leaves, respectively, than in the controls. The changes of CAT, but not POD or SOD activity were correlated to levels of aphid resistance. H_2_O_2_ levels were higher in local than in systemic leaves in contrast to CAT activity. *Tobacco curly shoot virus* mediated virus-induced gene silencing was employed to determine if CAT activation was involved in the aphid resistance induced by *B*. *tabaci* nymphs. *B*. *tabaci* induced CAT activity decreased when the *Cat1* expression was silenced. The performance assay indicated that *Cat1* silencing made *B*. *tabaci* infested plants a more suitable host for aphids than infested control plants. The aphid survival rate was reduced by 40.4% in infested control plants, but reduced by only 26.1% in *Cat1*-silenced plants compared to uninfested controls. Also, qPCR results showed that silencing of *Cat1* led to the suppression of the *B*. *tabaci* mediated *PR-2a* expression.

**Conclusions/Significance:**

Aphid resistance in plants infested with *B*. *tabaci* nymphs is associated with enhanced antioxidant activities in which CAT may play a dominant role. This resistance probably acted via interactions with SA-mediated defense responses.

## Introduction

Defense responses are often induced in terrestrial plants following attack by pathogens or insects. These responses may be localized in the damaged tissue or systemic in undamaged tissue. They may arise rapidly or with significant delay [1]. When attacked by pests, plants initiate sophisticated defense responses including the regulation of defense signal-transduction pathways and production of defensive compounds [2, 3], which can result in local or systemic resistance to subsequent infestations [4, 5].

The production of reactive oxygen species (ROS) in plants occurs early in plant defense response to external stimuli [6–8]. Superoxide (O_2_^−^) and hydrogen peroxide (H_2_O_2_) are the most important reactive oxygen species, and they rapidly accumulate in response to biotic stresses like herbivore feeding and pathogens or abiotic stresses such as high light intensity and cold stress [9]. However, high levels of ROS may damage the bodies of living organisms. To prevent oxidation burst due to ROS production, plants have evolved complex protective mechanisms for scavenging ROS. These include the enzymatic scavenging systems, such as catalase (CAT), peroxidase (POD), and superoxide dismutase (SOD) [10]. Catalase is a major H_2_O_2_-scavenging enzyme and functions mainly in the removal of excessive H_2_O_2_ generated during developmental or by environmental stimuli into water and oxygen in all aerobic organisms [11]. CAT is involved in the cell wall resistance of plants [6] and it also acts as a signal for the induction of defense genes [12]. CAT is generally composed of a multigene family and has been reported in many plant species, including *Arabidopsis* [13], barley [14], cotton [15], maize [16], sunflower [17], and tobacco [18]. In tobacco, there are three CAT isoforms (*Cat1*, *Cat2* and *Cat3*) identified [19], and *Cat1* is the predominant one in mature leaves [18]. Transgenic tobacco plants expressing antisense constructs of peroxisomal *Cat1* displayed severely reduced catalase activity and resistance under high light stress[20, 21]. SOD is able to catalyze the dismutation of O_2_^−^ to H_2_O_2_ and O_2_ [22, 23]; POD can catalyze the biosynthesis of lignin and suberin [10, 24].

Plants deploy defense-signaling pathways in response to biotic stress [25]. The salicylic acid (SA) pathway regulates the expression of a wide array of defense-responses including the pathogen related (PR) proteins and defense enzymes which have been shown to be important in both basal and resistance gene (R)-mediated biotrophic pathogen defense [26–28]. Recent evidence suggests that phloem-sucking hemipterans, such as aphids and whiteflies, are also associated with the salicylate responses [29, 30]. High levels of SA have been demonstrated to be involved in the defense response against aphids [31–34]. Also, a close relationship exists between the SA-mediated response and the cell redox state. In legume roots, inhibiting antioxidant enzyme activities would suppress expression of the pathogen induced *PR* gene [35].

The whitefly *Bemisia tabaci* Middle East-Asia Minor 1 (MEAM1) is an exotic sap-sucking pest that has broad host distributions [36, 37]. As an invasive species, *B*. *tabaci* faces competition from native phytophagous arthropods which are in the same niche and share a similar food range [38]. Recently, studies of *B*. *tabaci* and its herbivorous competitors *Trichoplusia ni*, *Pieris rapae*, *Liriomyza trifolii*, and *Trialeurodes vaporariorum* have shown that the presence of *B*. *tabaci* might influence the performance of these competitors via host plant induced defense reactions [39–42].

The interspecific competition among arthropods that share the same host plants may rely mostly on damage-induced plant reactions [43, 44]. Induction of antioxidant enzymes in plants due to herbivore damage has received significant attention [45, 46]. The enzymes CAT, POD, SOD play an important role in plant defense against different stresses, including insect herbivory [47–49]. He et al. [50] demonstrated that antioxidant enzymes contributed to chrysanthemum resistance against *Macrosiphoniella*. Research on *B*. *tabaci* induced modification of the antioxidant enzyme activities is relatively scarce. *B*. *tabaci* feeding can alter antioxidant enzyme activities in cabbage, cucumber and black gram plants [51–53], while the antioxidant enzyme responses of *B*. *tabaci* infested tobacco plants is not understood. Little research exists on the role of antioxidant enzymes in the interspecific competition among *B*. *tabaci* and other native herbivores, especially other phloem feeders. Zhang et al.[52], however, reported effects of antioxidant enzymes induced by *B*. *tabaci* on cabbage plants, on chewing caterpillar *Pieris rapae*.

In our preliminary research, *B*. *tabaci* nymph feeding induced resistance in tobacco against *M*. *persicae* [54], and this resistance was SA-depended[33, 34]. As the SA-mediated response is closely related to the cell redox state, we hypothesize that the antioxidant enzymes are involved in the plant defense response against aphids that is induced by *B*. *tabaci* nymphs. In this study, a plant-whitefly-aphid model was created, and the potential association between antioxidant enzymes activity level and this induced resistance was studied using spectrophotometry, virus induced gene silencing and quantitative real-time PCR techniques. We attempted to find the answers for the following questions: (1) What is the impact of *B*. *tabaci* nymph feeding on antioxidant enzyme levels in tobacco plants? (2) What antioxidant enzymes contribute to induced resistance against *M*. *persicae* in tobacco plants? (3) What is the relationship between the antioxidant enzymes and the SA-mediated defense responses?

## Materials and Methods

### Tobacco plants

Tobacco (*Nicotiana tabacum* L. variety *Xanthi*-nc) seeds were supplied by Nanjing Agricultural University, Institute of Plant Protection, Department of Plant Pathology, Nanjing, Jiangsu Province, China. Seeds were sown in plastic trays (50 cm × 25 cm) containing garden soil. The plants were cultivated in growth chambers at 23 ± 2°C and a relative humidity of 75 ± 5%. Plants with two leaves were individually transplanted into plastic pots (10 cm deep, 12 cm in diam) and placed in insect-proof screened cages (50 cm × 50 cm × 50 cm, 50 mesh). The plants were watered as needed and fertilized every 2 weeks at a rate of 0.05 g (N:P:K = 20:20:20) per plant and used for experiments when the plants had five leaves.

### Insect colonies

*Bemisia tabaci* Middle East-Asia Minor 1 (MEAM1) strain [55] was originally collected from cabbage and *M*. *persicae* was collected from tobacco plants grown at the Shandong Agricultural University Research Farm, Shandong Province, China. Insects were reared on tobacco plants for more than 30 generations. *B*. *tabaci* was identified based on the mitochondrial DNA COI gene sequence.

All of the pre-infestation and bioassay experiments were conducted in an artificial climate chamber (RTOP-D model, Top Instrument Corporation, Zhejiang, China) at 23 ± 2°C and 75 ± 5% RH (Relative humidity) and 3000 lx in continuous light, with a photoperiod of 12:12 (L:D) h.

### Whitefly infestation experiments

Pretreatment was performed using a method previously reported by Xue et al. [54]. Tobacco plants with five expanded leaves were placed in a nylon screen cage (50 cm × 50 cm × 50 cm) with ≈ 500 (± 10) whitefly adults (≈ 1:1 female:male ratio) per plant. After 4 h of feeding, adult whiteflies were removed by aspiration to synchronize egg hatching and nymphal development.

### Determination of Antioxidant Enzymes

Immature whitefly-infested plants were sampled at 5, 10, 15, and 20 d after the removal of adults, at which points the nymphs had reached the 1^st^, 2^nd^, 3^rd^, and 4^th^ instar, respectively. The fourth leaf, which had 9–10 nymphs/cm^2^, and the seventh leaf, which had none, were sampled as representative local and systemic leaves, respectively [34]. Leaves at the same positions on uninfested plants were sampled as controls. Before sampling, the insects on the local leaves were gently removed with a fine brush pen. Whole leaf was subsequently abscised from the plants, and the main veins were removed. Four plants each from the treatment group and the untreated control group were individually assayed for antioxidant enzymes. All spectrophotometric analyses were conducted in a Shimadzu UV-2450 spectrophotometer (Shimadzu, Arlington, MA, U.S) at room temperature.

Enzyme extract for catalase, peroxidase, and superoxide dismutase was prepared by first freezing the weighed amount of leaf samples (0.2 g) in liquid nitrogen to prevent proteolytic activity followed by grinding with 2 ml extraction buffer (0.1 M phosphate buffer, pH 7.5, containing 0.5 mM EDTA (Ethylenediaminetetraacetic acid) and 1 mM ascorbic acid). Brie was passed through four layers of cheesecloth and filtrate was centrifuged at 12,000 g for 10 min at 4°C and the supernatant was stored at -20°C until used in spectrophotometric assays of enzymes.

Catalase activity was measured as described by Aebi et al. [56]. Briefly, a decrease in absorbance (ABS) of 30 μM solution of H_2_O_2_ was monitored at 240 nm for 4 min. The results were presented as ABS240 variation per minute per gram fresh protein.

POD activity was measured according to Kar and Mishra et al. [57], an aliquot of 100 μl of leaf enzyme extract was added to 1 ml of 2.92 mM guaiacol (Sigma) with 0.02 mM H_2_O_2_ in phosphoric acid buffer (0.1 M, pH 8.0). The absorbance variation at 470 nm was measured for 4 min at 20-s intervals and the obtained value was calculated per 1 min. The results were presented as ABS470 variation per minute per gram fresh protein.

The estimation of SOD activity was performed with the procedure described by Beauchamp and Fridovich [58]. The reaction mixture contained 1.5 ml 0.05 M PBS (Phosphate buffer saline, pH 7.8), 0.3 ml 0.1 mM EDTA, 0.3 ml 0.13 M methionine, 0.3 ml 0.75 mM NBT (Nitrotetrazolium Blue chloride), 0.3 ml 0.02 mM riboflavin, and 0.1 ml enzymatic extract. After exposure to fluorescent light (4,000 lux) for 8 min, the absorbance variation was recorded at 560 nm. SOD activity was determined by its 50% inhibition of the NBT reduction caused by the superoxides generated from the reaction of photo-reduced riboflavin and oxygen. The results were expressed in units per milligram gram of protein.

### Determination of Hydrogen Peroxide

Experimental tobacco plants were obtained as described above. Hydrogen peroxide was estimated by forming a titanium—hydro peroxide complex [59]. Then, 1 g leaf material was grinded with 10 ml cooled acetone in a cold room (10°C). The mixture was filtered with Whatman No. 1 filter paper followed by the addition of 4 ml titanium reagent and 5 ml ammonium solution to precipitate the titanium—hydro peroxide complex. The reaction mixture was centrifuged at 10000×g for 10 min. Precipitate was dissolved in 10 ml 2 M H_2_SO_4_ and then recentrifuged. Supernatant was read at 415 nm against reagent blank in Shimadzu UV-2450 spectrophotometer (Shimadzu, Arlington, MA, U.S). The concentration of hydrogen peroxide was calculated from a standard curve. Four plants each from the treatment group and the untreated control group were individually assayed for hydrogen peroxide contents. The results were expressed in μmol H_2_O_2_ per gram fresh weight.

### Virus Induced Gene silencing

Total RNA was isolated from uninfected tobacco leaf using an RNA isolation kit (Takara Biotechnology, Dalian, China). The first-strand cDNA was synthesized from 1 μg of RNA using the HiScript 1st Strand cDNA Synthesis Kit (Vazyme Biotech, Nanjing, China). A 232-bp fragment spanning nucleotides of the *Cat1* gene (Genebank accession no.U93244.1) was amplified from the cDNA using a gene-specific primer pair si*Cat1* (S1 Table). The resulting PCR product was cloned into *XbaI-BamHI*-digested pBIN2mDNA1 plasmid to generate the gene-silencing vector 2mDNA1- *Cat1* according to the method of Luan et al. [60]. After the gene-silencing vector was sequenced to confirm the fidelity of the inserts, it was transformed into *A*. *tumefaciens* strain EHA105 by electroporation. *Tobacco curly shoot virus* (TbCSV) was used as a helper virus in the VIGS assay. Approximately, 0.2 mL of *A*. *tumefaciens* cultures (ABS600 = 0.6) carrying TbCSV and 2mDNA1-*Cat1* constructs were co-infiltrated into the stem of each plant at the three-to-four true-leaf stage (VIGS silenced plants). Control, empty-vector plants were inoculated with *A*. *tumefaciens* cultures carrying TbCSV and pBIN2mDNA1 (without the gene insert). All plants were grown in a greenhouse under the same conditions as described above, and cultivated to the four to five true-leaf stage. Prior to use of the VIGS-silenced plants, total RNA was isolated from the second leaf from the top. *Cat1* RNA levels in the VIGS-silenced plants were determined by qRT-PCR using the *qCat1* primer pair to determine the efficiency of silencing. The *Actin* gene served as the endogenous control (S1 Table). VIGS-silenced and empty-vector plants were used for whitefly performance studies (below).

### Quantitative Real-time PCR

Experimental tobacco plants were obtained as described above. After 15 d, the local (fourth leaf) damaged leaves, systemic (seventh leaf) leaves from infested and uninfested tobacco plants (the last of which served as controls) were sampled for analysis of defense gene expression. Total RNA samples were isolated. cDNA was synthesized using the SYBR^®^ PrimeScript RT-PCR Kit II (Takara Biotechnology, Dalian, China). *PR-2a* (Genebank accession no.M59443.1) and *Cat1* (Genebank accession no.U93244.1) RNA levels were measured using qRT-PCR. qRT-PCRs were performed using the Bio-rad CFX96 Real-Time PCR System (Bio-Rad Laboratories, Hercules City, USA) with SYBR-Green detection. Each gene was analyzed in triplicate in each of three biologically independent treatments. The average threshold cycle (Ct) was calculated per sample. The relative expression levels were calculated with the 2^-ΔΔCT^ method. The reference gene *Actin* (Genebank accession no.X69885.1) was used for transcript normalization. Primers for qRT-PCRs and the list of accession numbers are provided in S1 Table.

### Survival of aphids on whitefly pre-infested tobacco plants

Experimental tobacco plants were obtained as described above. Plants (*Cat1*-slienced and empty vector injected) infested by whitefly nymphs were used after the nymphs reached the third instar (≈ 15 d). At this time plants had developed to seven to eight fully expanded leaves. The fourth leaves with 9–10 nymphs/cm^2^ and seventh leaves without nymphs served as local and systemic leaves, respectively

Fifteen adult apterous *M*. *persicae* were placed on the lower surfaces of local (fourth leaf) and systemic (seventh leaf) leaves of pre-infested and uninfested plants and confined using a clip-on leaf cage (2 cm deep, 6 cm in diam). They were allowed to reproduce for 24 h and were then removed using a small soft brush, and 20 newborn nymphs were caged on the same leaf. Survival of *M*. *persicae* was monitored daily until they matured and started reproducing offspring, generally on day 8 (the first generation). Survival was calculated from the average number of aphids per plant. Each treatment had 10 replications.

## Statistical analysis

Data were analyzed using the statistical software package SPSS version 18.0 (SPSS, Chicago, USA). *M*. *persicae* survival was tested with a Cox proportional hazards model. Significant differences in the antioxidant enzyme levels and H_2_O_2_ content of tobacco leaves were tested by a mixed effect model and factorial ANOVA model using Tukey’s test at a significance level of 5% (*P* < 0.05).

## Results

### Antioxidant enzyme activity levels on tobacco plants infested with whitefly nymphs

**Fig 1:**
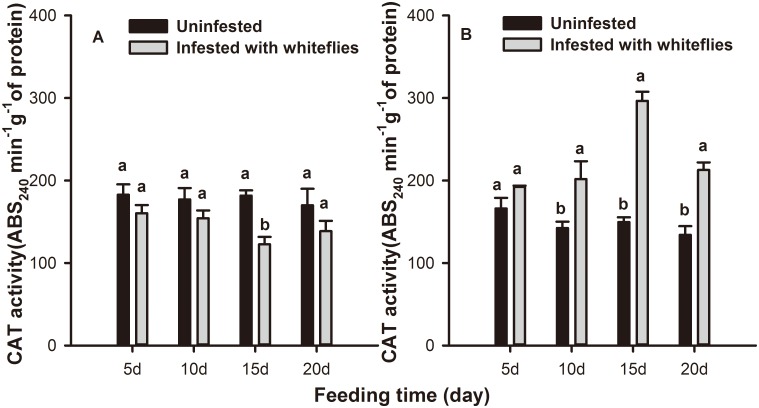
CAT activity levels in tobacco plants infested with *B*. *tabaci* nymphs. CAT activity levels in (A) local and (B) systemic leaves of tobacco plants after 5, 10, 15, and 20 days of *B*. *tabaci* nymphs infestation. Values represent the mean ABS variation per min per g protein ± standard error. Paired means with the same letter are not significantly different (P > 0.05).

The antioxidant enzymes were differently affected by *B*. *tabaci* nymph infestation. Whitefly infestation (*F* = 4.343; *df* = 1,58; *P* = 0.042) and leaf position (*F* = 6.083; *df* = 1,58; *P* = 0.017) but not feeding time (*F* = 0.973; *df* = 3,58; *P* = 0.429) had significant effects on CAT activity in tobacco leaves in the mixed effects model. The activities of CAT in local and systemic leaves of *B*. *tabaci* infested plants were not significantly different from controls until 15 d, when CAT activity was significantly higher than controls by 98.2% systemically (*F* = 21.233; *df* = 1,31; *P* = 0.000), while 32.6% lower than in controls locally (*F* = 3.072; *df* = 1,31; *P* = 0.042) (Fig 1A & 1B).

**Fig 2:**
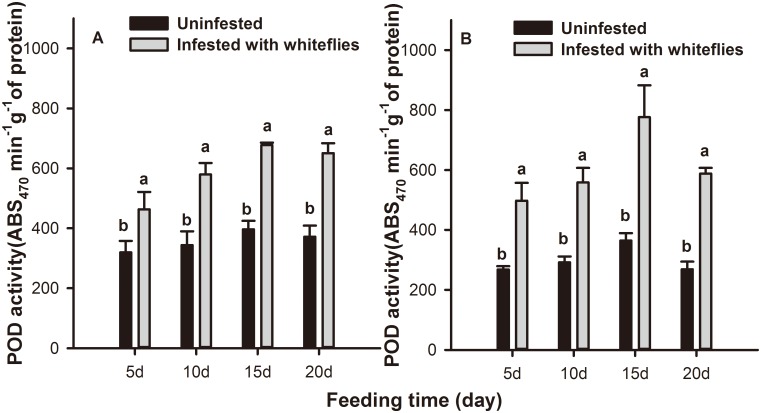
POD activity levels in tobacco plants infested with *B*. *tabaci* nymphs. POD activity levels in (A) local and (B) systemic leaves of tobacco plants after 5, 10, 15, and 20 days of *B*. *tabaci* nymph infestation. Values represent the mean ABS variation per min per g protein ± standard error. Paired means with the same letter are not significantly different (P > 0.05).

Whitefly infestation (*F* = 141.127; *df* = 1,58; *P* = 0.000), feeding time (*F* = 9.245; *df* = 3,58; *P* = 0.000) but not leaf position (*F* = 1.073; *df* = 1,58; *P* = 0.305), had significant effects on POD activity in tobacco leaves. Significantly higher levels of POD activity were detected in local and systemic leaves of *B*. *tabaci* nymph infested plants than in uninfested control plants during the entire feeding period. At 15 d, *B*. *tabaci* nymph infestation rendered the activity of POD 71.0% higher than in controls locally (*F* = 14.137; *df* = 1,31; *P* = 0.000) and 112.9% higher than in controls systemically (*F* = 13.770; *df* = 1,31; *P* = 0.001) (Fig 2A & 2B).

**Fig 3:**
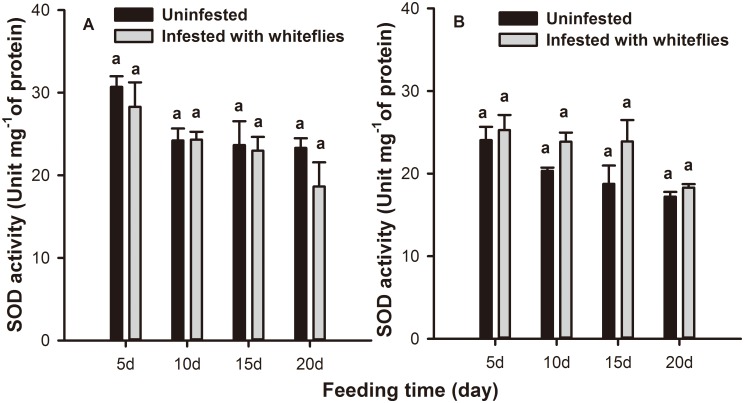
SOD activity levels in tobacco plants infested with *B*. *tabaci* nymphs. SOD activity levels in (A) local and (B) systemic leaves of tobacco plants after 5, 10, 15, and 20 days of *B*. *tabaci* nymphs infestation. Values represent the mean unit per mg protein ± standard error. Paired means with the same letter are not significantly different (P > 0.05).

Feeding time (*F* = 11.635; *df* = 3,58; *P* = 0.000) and leaf position (*F* = 10.707; *df* = 1,58; *P* = 0.002) but not whitefly infestation (*F* = 0.099; *df* = 1,58; *P* = 0.755) had significant effects on SOD activity in tobacco leaves. No significant difference in SOD activity was detected in either local or systemic leaves during the whole feeding period (Fig 3A & 3B).

### Quantification of H_2_O_2_ levels in plants infested with whitefly nymphs

**Fig 4:**
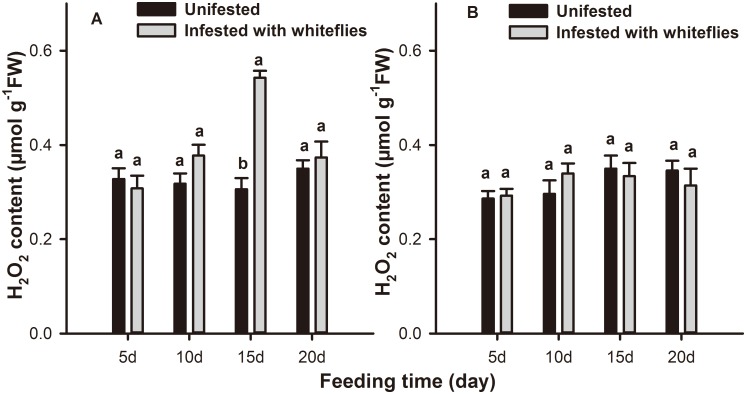
Quantification of H_2_O_2_ levels in *B*. *tabaci* nymph-infested tobacco plants. H_2_O_2_ levels in (A) local and (B) systemic leaves of tobacco plants after 5, 10, 15, and 20 days of *B*. *tabaci* nymphs infestation. Values represent the mean μmol H_2_O_2_ per g fresh weight (FW) ± standard error. Paired means with the same letter are not significantly different (P > 0.05).

There were significant effects of whitefly infestation (*F* = 4.262; *df* = 1,58; *P* = 0.043), feeding time (*F* = 3.247; *df* = 3,58; *P* = 0.028) and leaf position (*F* = 11.701; *df* = 1,58; *P* = 0.021) on H_2_O_2_ levels of tobacco leaves. In local leaves, the H_2_O_2_ concentration was not significantly different from the controls until 15 d after *B*. *tabaci* nymphs infestation, at which time the H_2_O_2_ level was 77.3% (*F* = 6.204; *df* = 1,31; *P* = 0.000) higher than control (Fig 4A). The H_2_O_2_ content on systemic leaves of infested plants did not differ significantly from that of the uninfested control (Fig 4B).

### *Cat1* expression and CAT activity level

**Fig 5:**
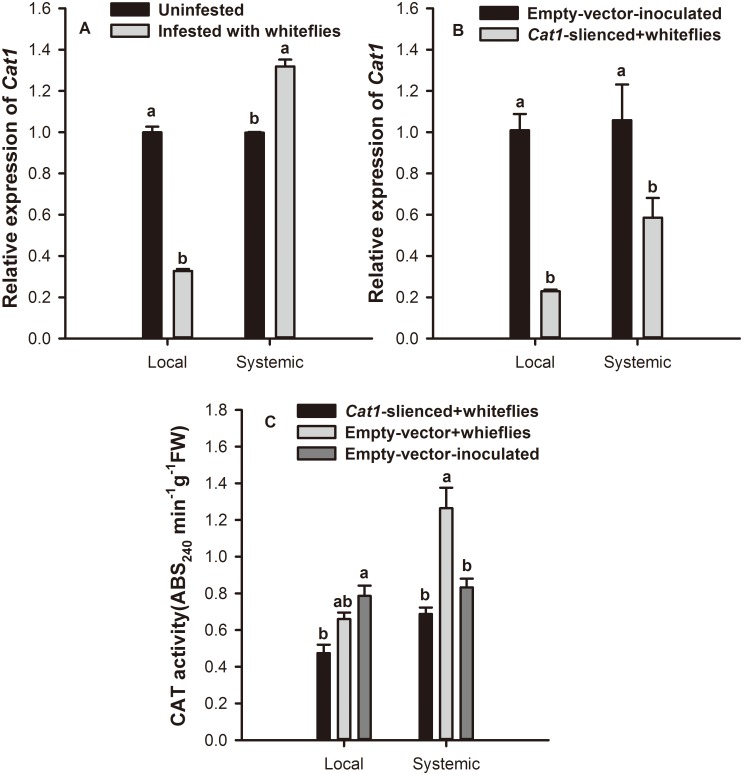
Infestation by *B*. *tabaci* nymphs on relative expression of *Cat1* in tobacco plants. Relative expression of *Cat1* on systemic and local leaves of (A) empty vector injected and (B) *Cat1*-silenced plants. Values represent the mean expression levels of *Cat1* ± standard error. (C) CAT activity on systemic and local leaves of *Cat1*-silenced tobacco plants. Paired means with the same letter are not significantly different (P > 0.05).

*B*. *tabaci* nymph infestation significantly increased expression of *Cat1* in systemic leaves while inhibiting expression in local leaves of empty vector control plants. Expression was 1.32-fold (*F* = 180.846; *df* = 1,11; *P* = 0.000) and 0.33-fold (*F* = 180.846; *df* = 1,11; *P* = 0.000) that of control, respectively (Fig 5A). In *Cat1*-silenced plants, expression of *Cat1* was significantly suppressed by the infestation of *B*. *tabaci* nymphs. It was only 0.22-fold (*F* = 7.676; *df* = 1,11; *P* = 0.004) and 0.58-fold (*F* = 7.676; *df* = 1,11; *P* = 0.045) that of control in local and systemic leaves, respectively (Fig 5B).

CAT activity was 51.8% higher in the systemic leaves of empty vector plants after *B*. *tabaci* nymph infestation (*F* = 12.340; *df* = 2,23; *P* = 0.001) compared to the uninfested control. Meanwhile, no significant difference in CAT activity was found between infested *Cat1*-silenced plants and uninfested control plants. In local leaves, CAT activity was 39.7% lower in infested *Cat1*-silenced plant than in uninfested control (*F* = 12.340; *df* = 2,23; *P* = 0.009) (Fig 5C).

### The relationship between CAT and aphids survival

**Fig 6:**
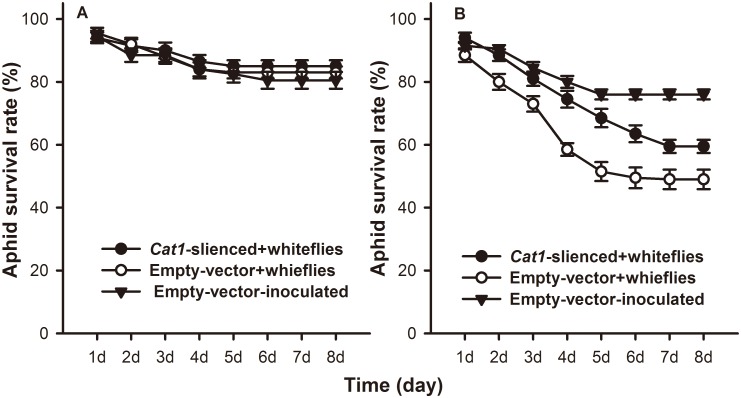
*M*. *persicae* survival on *B*. *tabaci* nymph-infested plants. *M*. *persicae* survival on (A) local and (B) systemic leaves of *B*. *tabaci* nymph-infested tobacco plants. Values represent the mean survival of aphids ± standard error.

Based on the Cox proportional hazards model, survival of *M*. *persicae* on local leaves of either infested *Cat1*-silenced or empty vector injected plants did not differ significantly from that of the uninfested control (*P* = 0.736 and 0.822, respectively) (Fig 6A). Aphids survival was 40.4% (*F* = 24.968; *df* = 2,29; *P* = 0.000) lower than the uninfested control on systemic leaves of *B*. *tabaci* nymph infested empty vector injected plants, while only 26.1% (*F* = 24.968; *df* = 2,29; *P* = 0.000) lower than uninfested control on *Cat1*-silenced plants (Fig 6B).

### The relationship between CAT and *PR* gene expression

**Fig 7:**
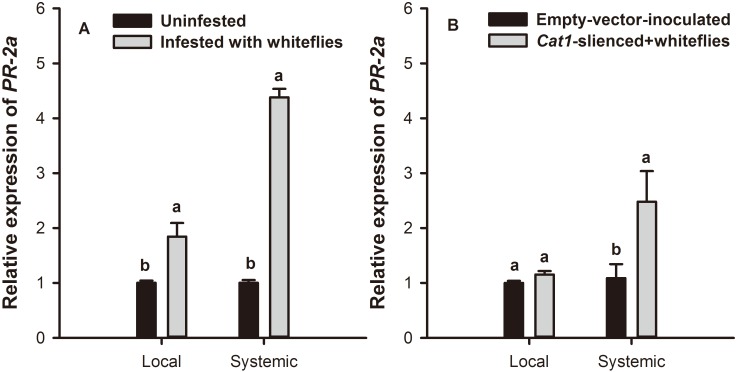
Relative expression of *PR-2a* on *B*. *tabaci* nymph-infested tobacco plants. Relative expression of *PR-2a* on local and systemic leaves of (A) empty vector injected and (B) *Cat1*-silienced tobacco plants. Values represent the mean *PR* gene expression levels ± standard error. Paired means with the same letter are not significantly different (P > 0.05).

*B*. *tabaci* nymph feeding significantly increased the expression of *PR-2a*. Expression levels in systemic leaves of wild-type plants was 1.84-fold (*F* = 62.244; *df* = 1,11; *P* = 0.018) and 4.38-fold (*F* = 62.244; *df* = 1,11; *P* = 0.000) that of the control in local and systemic leaves, respectively (Fig 7A). In *Cat1*-silenced plants, it was 1.15-fold (*F* = 4.034; *df* = 1,11; *P* = 0.768) and 2.48-fold (*F* = 4.034; *df* = 1,11; *P* = 0.020) that of the control in local and systemic leaves, respectively (Fig 7B).

## Discussion

Plants generate many inducible defense responses to herbivore attack [61]. Induced plant resistance could be correlated with the redox status of the host tissue, which is achieved by the generation of ROS and their subsequent elimination [35, 62]. CAT, SOD, and POD are the major enzymes of the cleaning system of ROS and they could effectively inhibit ROS from damaging the bodies of living organisms, and play a significant role in the induced defense response [48, 49, 51]. In our biochemical assay, antioxidative enzyme activity levels varied with leaf position and the feeding time of the *B*. *tabaci* nymphs. At 15 d following infestation, CAT activity was significantly increased in systemic leaves while it was suppressed in local leaves compared to the controls. This trend is consistent with that of the aphid resistance observed in our previous data, i.e., that a *B*. *tabaci* infestation could decrease the performance of aphids on systemic but not on local leaves [34], and that resistance to aphids induced by *B*. *tabaci* was most obvious at the third instar [63]. These data indicated that CAT was considered to be involved in the defense response against aphids. *B*. *tabaci* infestation caused an increase in POD but not SOD activity. However, POD activity was significantly higher than controls in both local and systemic leaves at 15 d. The trends of SOD and POD activity were inconsistent with that of the aphid performance results. Therefore, POD and SOD may not be involved in aphid defense. Furthermore, at 15 d after *B*. *tabaci* infestation, the changing trends of H_2_O_2_ levels was opposite that of CAT activity indicating that H_2_O_2_ might also be unrelated to aphids resistance despite data demonstrating that it can cause oxidative damage to midgut cells and impair the nutrient absorption of herbivores [64]. In general, the accumulation of H_2_O_2_ resulted from the catalytic action of SOD while, in the test model, the level of SOD activity was consistent during the entire feeding period; the special components may spur this phenomenon and the mechanism will be explored in future research.

Catalase is a significant component of the cell protective mechanism against oxidative stress [11, 65, 66], and it plays a crucial role in maintaining the induced defense response [6, 12]. For instance, alfalfa plants resistant to the spotted alfalfa aphid contained higher activities of CAT than susceptible plants [67], and *Acyrthosiphon pisum* feeding on artificial diets with high CAT protein concentrations experienced decreased performance [68]. In tobacco, *Cat1* was the predominant gene in mature leaves [18]. Our qRT-PCR assay demonstrated that the trend of *Cat1* relative expression was consistent with that of CAT activity in infested control plants, and *B*. *tabaci* induced CAT activity decreased when virus-induced gene silencing was utilized to suppress the *Cat1* expression. These findings are in line with previous studies showing that transgenic tobacco plants expressing an antisense construct of peroxisomal *Cat1* displayed severely reduced catalase activity [69]. Bioassay results showed that *B*. *tabaci* could significantly inhibit the survival of aphids on control plants. However this negative effect on aphids infestation was reduced to some extent by suppressing the expression of *Cat1*. In this way, CAT may contribute to the aphid resistance.

Many studies have found that SA is involved in resistance of certain plants to phloem-feeding aphids. For example, activating the SA signal pathway may decrease the performance of aphids on potato plants, and application of Benzothiazole (BTH) reduces aphid growth [70–72]. Our prior evidence also suggests that a close association exists between *B*. *tabaci* induced aphid resistance and the SA signal pathway [33]. Redox processes may also be involved in SA-mediated defensive signal transduction and elicitation. For instance, expression of the antisense *CAT* gene, as well as the CAT inhibitor 3-amino-1,2,4-triazole showed reduced expression of the *PR* gene in legume roots [35]. In the present study, *B*. *tabaci* infestation caused increased expression *PR-2a* gene in both local and systemic leaves of tobacco plants, and the expression levels were much higher in systemic than in local leaves. This trends was opposite our previous results that local leaves had higher SA levels than systemic leaves in *B*. *tabaci* infested tobacco plants [34], and indicates that the SA-mediated defense response was suppressed in local leaves. Interestingly, virus-induced gene silencing of *Cat1* led to the suppression of the *B*. *tabaci* mediated *PR-2a* expression. In this way, there should be a positive correlation between the expression of *Cat1* and SA-mediated defense genes. It is possible that feeding of *B*. *tabaci* changed the redox status in tissues of the systemic leaves to a reducing status via inducing CAT activity. In reducing status, the NPR1 oligomer could translocate to the nucleus and activate the expression of a battery of *PR* genes [73]. In contrast, in local leaves, *B*. *tabaci* infestation suppressed CAT activity resulting in accumulation of H_2_O_2_ which inhibits the defense responses. However, other studies have found H_2_O_2_ levels to be positively associated with SA-mediated defense responses [74, 75]. This may be because that interactions between H_2_O_2_ and SA are multi-faceted, and can vary from cooperation to mutual inhibition in different contexts, concentrations and conditions [76]. The *PR-2a* gene may encode β-1,3-glucanase which could release oligosaccharides from the plant cell wall; these are molecules that are known to trigger other defense reactions in plants [77, 78]. It has also been demonstrated that β-1,3-glucanase is involved in plant defense against pests and pathogens [34, 39, 79].

Previous research indicates that the defense responses and adverse impact of different leaf positions of plants pre-infested with phloem-feeders or other herbivores are variable [80, 81]. Zhao et al. [34] found β-1,3-glucanase and chitinase activities were much higher in systemic than in local leaves of *B*. *tabaci* infested tobacco plants. In the present study, CAT activity was increased in systemic leaves but suppressed in local leaves after *B*. *tabaci* nymphs infestation. Insect saliva also plays an important role in plant induced defense [82]. For example, a transcript encoding phloem protein 2 lectin (PP2-A7), proposed to function in aphid defense, had a 5-fold higher expression level in infested leaves and a 22-fold higher expression level in systemic leaves than equivalent control in *Arabidopsis* [75]. We speculate that there may be specific components in *B*. *tabaci* saliva that weaken the local host defense. Similarly, *Helicoverpa* (Lepidoptera:Noctuidae) saliva catalyzes the generation of H_2_O_2_ that suppresses induced defenses in *N*. *tabacum* plants [83]. Other research suggests that high levels of SA cause inhibition of CAT activity [12]. In this study, inhibition of CAT activity in local leaves may be unrelated to increased SA levels, as *B*. *tabaci* infestation significantly increased CAT activity in systemic leaves as it did for SA levels in our previous research [34]. Moreover, Bi et al. [64] and Kim et al. [84] indicated that application of SA will not decrease the CAT levels in tobacco plants.

Redox processes may be involved in chemical mediation of interspecific competition among herbivores sharing the same food plant [62]. ROS production and scavenging are intimately linked, and the balance between them will determine the defense signaling output [35]. The induced defense response helps to improve the competitive ability of *B*. *tabaci* to displace other herbivores. [52, 54]. The data from this study supports our hypothesis that induced antioxidant responses are important components of plant-herbivore relationships. The antioxidant enzyme CAT plays a crucial role in *B*. *tabaci* induced defense response against aphids. It may mediate redox-sensitive defense elements in SA signal pathway by altering the redox status in tissues of infested plants, and then influence aphid resistance. However, more further works were needed to prove this.

## Supporting Information

S1 Table Information on genes tested in this study. (DOC)

## Abbreviations

RH: Relative humidity
CAT: Catalase
POD: Peroxidase
SOD: Superoxide dismutase
ROS: Reactive oxygen species
ABS: Absorbance
NBT: Nitrotetrazolium Blue chloride
PBS: Phosphate buffer saline
EDTA: Ethylenediaminetetraacetic acid
VIGS: Virus induced gene silencing
TbCSV: *Tobacco curly shoot virus*
BTH: Benzothiazole
NPR1: Nonexpressor of pathogenesis related genes1
