# Supplementary material for: Antioxidant Enzyme Responses Induced by Whiteflies in Tobacco Plants in Defense against Aphids: Catalase May Play a Dominant Role
Source: PLoS One. 2016 Oct 27;11(10):e0165454. doi: 10.1371/journal.pone.0165454 (PMC5082799; doi:10.1371/journal.pone.0165454)
Supplement: S1 Table — (DOC) [file pone.0165454.s001.doc]

**Table S1. Information on genes tested in this study**

| **Gene** | **Accession numbers** | **Primers** | **Tests** |
| --- | --- | --- | --- |
| *Actin* | X69885.1 | 5’-GCTTGCTTACATTGCTCTCGACTAT-3’  5’-GATAGAGTTGTATGTAGTCTCGTG-3’ | Real-time  RT-PCR |
| *qCat1* | U93244.1 | 5’-GGAGGAGGTGATGTCAATA-3’,  5’-CTGTAACTTCCATTGTTTCAG-3’ | Real-time  RT-PCR |
| *SiCat1* | U93244.1 | 5’-CG**GGATCC**CCTTGTCTGATCCTCGTA-3’ (***BamH* I**),  5’-GC**TCTAGA**TTTGATCGCCACCACATT-3’  (***Xba* I**), | VIGS |
| *PR2* | M59443.1 | 5’-TAACCTTCCACTCTTAGCCAATG-3’  5’-GCCAGCCACTTTCAGATACAATA-3’ | Real-time  RT-PCR |
